# Supplementary material for: Evidence of DISC1 as an arsenic binding protein and implications regarding its role as a translational activator
Source: Front Mol Biosci. 2023 Dec 7;10:1308693. doi: 10.3389/fmolb.2023.1308693 (PMC10773898; doi:10.3389/fmolb.2023.1308693)
Supplement: Supplementary file 1 [file DataSheet1.PDF]

Supplementary Material

Supplementary Figures

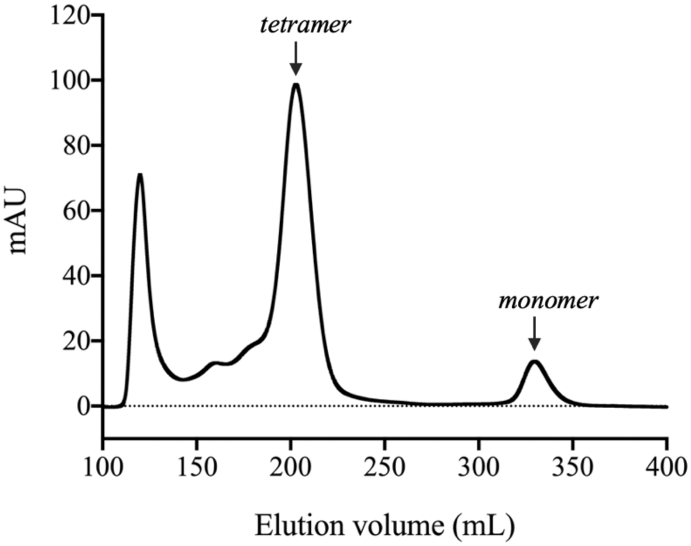

Figure S1. Size-exclusion chromatography elution profile obtained for the DISC1 C-terminal construct (WT) highlighting the peaks corresponding to monomeric and tetrameric species. The peak eluting between 100-150 mL contains high molecular weight oligomers and amorphous aggregates.

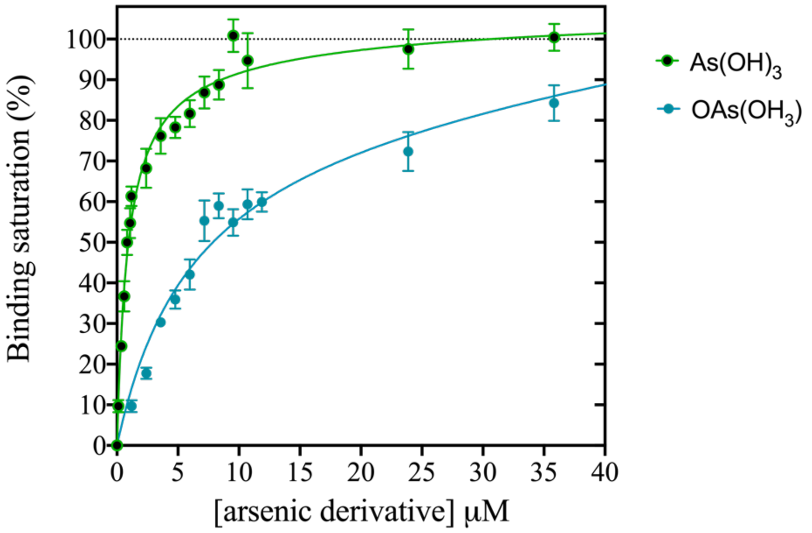

Figure S2. Binding saturation curves measured for WT in the presence of either trivalent arsenous acid ($As(OH_3)$, in green) or pentavalent arsenate ($OAs(OH_3)$, cyan).

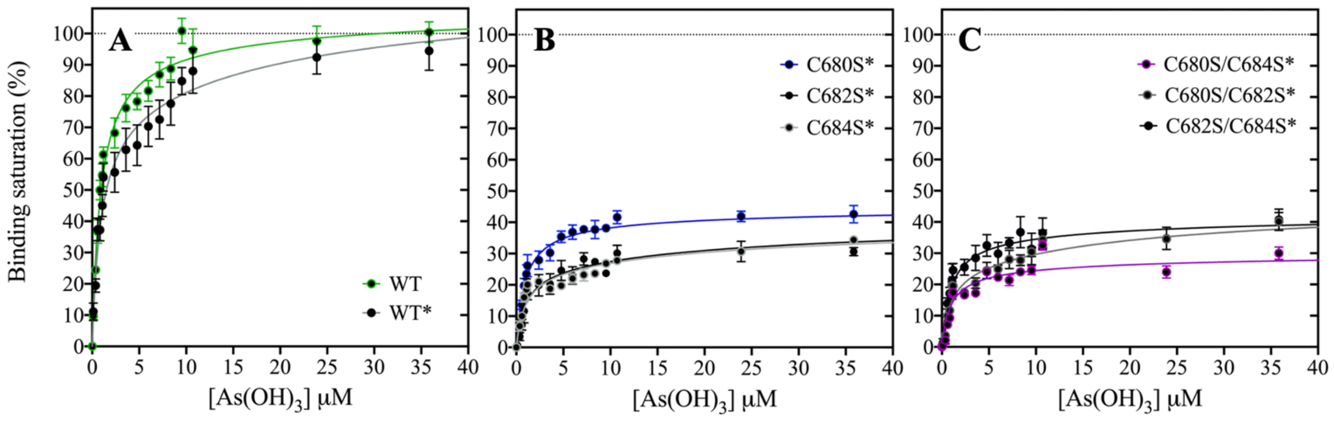

Figure S3. Plots of depicting the binding saturation as a function of arsenous acid concentration obtained for (**A**) WT and WT*, (**B**) the single cysteine variants C680S*, C682S*, and C684S*, and (**C**) the double mutants C680S/C682S*, C680S/C684S*, and C682S/C684S*. Lines correspond to a collective fit of all data presented in Table 1 using a simple two-site specific binding model.
